# Master Sculptor at Work: Enteropathogenic Escherichia coli Infection Uniquely Modifies Mitochondrial Proteolysis during Its Control of Human Cell Death

**DOI:** 10.1128/mSystems.00283-20

**Published:** 2020-06-02

**Authors:** Natalie C. Marshall, Maichael Thejoe, Theo Klein, Antonio Serapio-Palacios, Andrew S. Santos, Niklas von Krosigk, Jayachandran N. Kizhakkedathu, Nikolay Stoynov, Leonard J. Foster, Christopher M. Overall, B. Brett Finlay

**Affiliations:** a Department of Microbiology & Immunology, University of British Columbia, Vancouver, Canada; b Michael Smith Laboratories, University of British Columbia, Vancouver, Canada; c Life Sciences Institute and Department of Oral Biological & Medical Sciences, Faculty of Dentistry, University of British Columbia, Vancouver, Canada; d Department of Pathology and Laboratory Medicine, University of British Columbia, Vancouver, Canada; e Department of Biochemistry & Molecular Biology, University of British Columbia, Vancouver, Canada; California State University, Fresno

**Keywords:** infection, mitochondria, EPEC, type III secretion system, apoptosis, proteomics, SILAC, TAILS, N termini, proteolysis

## Abstract

Enteropathogenic Escherichia coli (EPEC) causes severe diarrheal disease and is present globally. EPEC virulence requires a bacterial type III secretion system to inject >20 effector proteins into human intestinal cells. Three effectors travel to mitochondria and modulate apoptosis; however, the mechanisms by which effectors control apoptosis from within mitochondria are unknown. To identify and quantify global changes in mitochondrial proteolysis during infection, we applied the mitochondrial terminal proteomics technique mitochondrial stable isotope labeling by amino acids in cell culture-terminal amine isotopic labeling of substrates (MS-TAILS). MS-TAILS identified 1,695 amino N-terminal peptides from 1,060 unique proteins and 390 N-terminal peptides from 215 mitochondrial proteins at a false discovery rate of 0.01. Infection modified 230 cellular and 40 mitochondrial proteins, generating 27 cleaved mitochondrial neo-N termini, demonstrating altered proteolytic processing within mitochondria. To distinguish proteolytic events specific to EPEC from those of canonical apoptosis, we compared mitochondrial changes during infection with those reported from chemically induced apoptosis. During infection, fewer than half of all mitochondrial cleavages were previously described for canonical apoptosis, and we identified nine mitochondrial proteolytic sites not previously reported, including several in proteins with an annotated role in apoptosis, although none occurred at canonical Asp-Glu-Val-Asp (DEVD) sites associated with caspase cleavage. The identification and quantification of novel neo-N termini evidences the involvement of noncaspase human or EPEC protease(s) resulting from mitochondrial-targeting effectors that modulate cell death upon infection. All proteomics data are available via ProteomeXchange with identifier PXD016994.

**IMPORTANCE** To our knowledge, this is the first study of the mitochondrial proteome or N-terminome during bacterial infection. Identified cleavage sites that had not been previously reported in the mitochondrial N-terminome and that were not generated in canonical apoptosis revealed a pathogen-specific strategy to control human cell apoptosis. These data inform new mechanisms of virulence factors targeting mitochondria and apoptosis during infection and highlight how enteropathogenic Escherichia coli (EPEC) manipulates human cell death pathways during infection, including candidate substrates of an EPEC protease within mitochondria. This understanding informs the development of new antivirulence strategies against the many human pathogens that target mitochondria during infection. Therefore, mitochondrial stable isotope labeling by amino acids in cell culture-terminal amine isotopic labeling of substrates (MS-TAILS) is useful for studying other pathogens targeting human cell compartments.

## INTRODUCTION

Apoptosis is a broadly conserved cell death process that removes damaged cells to protect tissues as a whole (1). In humans, this is essential in immune defense and healthy tissue maintenance, such as turnover of the intestinal epithelium (2, 3). Apoptosis can be triggered by various stimuli, from infection to irradiation, with all pathways converging at mitochondria. In healthy cells, mitochondria sequester highly toxic compounds, such as cytochrome *c*, which are released during apoptosis and activate cytosolic caspases that canonically cause cell-wide proteolysis and destruction (reviewed by Galluzzi et al. [4]). While best known as the “powerhouse of the cell,” mitochondria are also signaling hubs for many cellular processes, including the intrinsic (or “mitochondrial”) pathway of apoptosis and innate immunity (reviewed by Mills et al. [5]). Mitochondria are, therefore, appealing targets for pathogens because many human pathogens target mitochondria and regulate cell death to favor ongoing infection, including prominent human pathogens, such as Salmonella enterica, Neisseria meningitidis, and pathogenic Escherichia coli (reviewed by Rudel et al. [6]).

Enteropathogenic E. coli (EPEC) uses a type III secretion system to inject >20 virulence factors into infected human intestinal epithelial cells. Several type III-secreted (T3S) effector proteins localize to mitochondria and affect several steps in the intrinsic apoptosis pathway, including depolarization of inner mitochondrial membrane potential (ΔΨ_m_), release of cytochrome *c*, and activation of early and late stage caspase-9 and -3, respectively (7–10). For example, the conserved T3S effectors EspF and EspZ induce and delay intrinsic apoptosis during infection, respectively; both are essential for optimal bacterial colonization (7, 11, 12). The amino (N) terminus of EspF mimics a cleavable human mitochondrial targeting sequence (MTS) and, thus, hijacks human pathways for import into the mitochondrial matrix, at which point the MTS is cleaved (8). EspF localization to mitochondria is important for virulence; in a mouse model comparing full-length EspF with a mutant lacking the MTS (thereby preventing mitochondrial localization), infected mice had less intestinal inflammation, cell death, and mortality (7, 13). In contrast, EspZ interacts with the inner mitochondrial membrane to stabilize ΔΨ_m_ and, thereby, delay apoptosis (10); EspZ is important for cell death and colonization in a rabbit model and protects human intestinal cells from chemically induced apoptosis (12, 14).

Because EPEC mitochondrial-targeting effectors have opposing effects on cell death, studies examining mitochondria as a system during infection will inform on the net balance. However, our understanding of how EPEC controls apoptosis has been limited by the lack of techniques able to detect global mitochondrial changes. In particular, global proteolysis—a canonical aspect of apoptosis—has not been well characterized during infection (reviewed by Marshall et al. [15]). Recently, we addressed this gap by developing an N-terminal proteomics (terminomics) technique called mitochondrial stable isotope labeling by amino acids in cell culture-terminal amine isotopic labeling of substrates (MS-TAILS) to simultaneously assess global proteolysis within enriched mitochondria and whole cells in parallel (16). This technique is performed by detecting and quantifying the neo-N termini that are generated at the sites of proteolytic processing (17, 18). Previously, we reported the development of MS-TAILS to identify the normal mitochondrial N-terminome in healthy cells (16). We also directly compared cell lysates and their mitochondria from healthy cells with those from two models of chemically induced early intrinsic apoptosis by applying stable isotopic labeling between conditions. Here, we hypothesized that the identification and quantification of T3S-mediated changes in mitochondria would reveal how EPEC effectors control apoptosis during infection. Therefore, we applied MS-TAILS to study EPEC infection and identify mitochondrial changes that were distinct from apoptosis and, therefore, mediated directly or indirectly by mitochondrial-targeted T3S effectors.

## RESULTS

### EPEC infection and T3S effectors alter human mitochondrial membrane potential.

**FIG 1:**
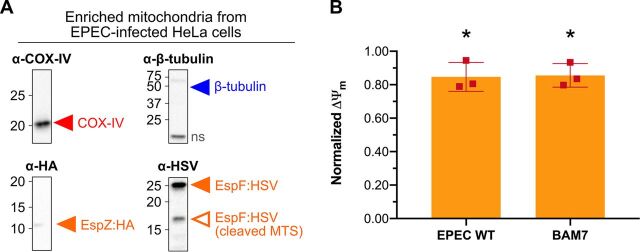
EPEC infection induced early intrinsic apoptosis with T3S effectors detected from enriched mitochondria. (A) HeLa cells were infected with EPEC strains containing C-terminally tagged EspZ and EspF. At 2 h postinfection, mitochondria were enriched and analyzed by SDS-PAGE and Western immunoblot using antibodies specific to each epitope tag (HA, α-hemagglutinin; HSV, α-herpes simplex virus) to detect EspZ:HA and EspF:HSV and markers of mitochondria (COX-IV, cytochrome *c* oxidase IV) and cytoplasm (β-tubulin) to assess mitochondrial purity. ns, nonspecific binding. (B) HeLa cells were infected with EPEC wild type (WT), or intrinsic apoptosis was induced by adding BAM7. Control conditions were prepared under the same conditions, either a mock infection or the addition of only the vehicle control (i.e., DMSO), respectively. Mitochondrial membrane potential (ΔΨ_m_) was measured using JC-1 dye, and data were normalized relative to each respective control condition. An unpaired, two-tailed *t* test was used to compare each experimental treatment with its respective control. *, *P < *0.05.

**FIG 2:**
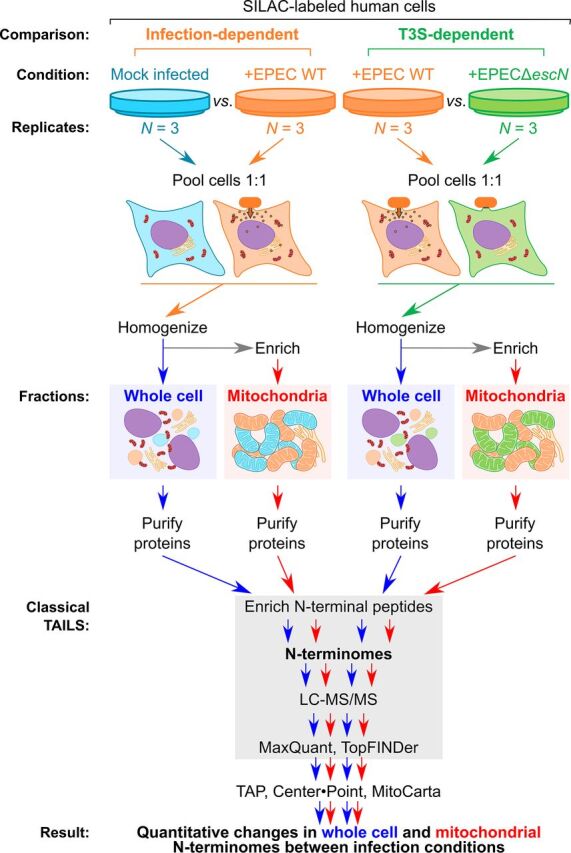
MS-TAILS experimental workflow. Light- or heavy-labeled HeLa cells were either mock infected, infected with EPEC wild type (WT; *n* = 3), or infected with EPEC Δ*escN* (*n* = 3), which cannot deliver T3S effectors. Cells were pooled 1:1 and homogenized. An aliquot was taken for parallel whole-cell TAILS analysis, and mitochondria were enriched from the remaining cell homogenates. Proteomes were purified from each fraction, and N-terminal peptides were enriched. Each N-terminal peptide was identified, quantified, and located within its full-length protein using tandem mass spectrometry and a bioinformatics pipeline.

EPEC infection conditions were optimized to capture the early events of type III-secreted (T3S) effectors on apoptosis when ΔΨ_m_ decreases as a sign of mitochondrial membrane depolarization before activation of the late-stage executioner caspase-3. To identify when mitochondrial-targeting effectors were present in human mitochondria during infection, mutant EPEC strains containing C-terminal epitope tags on full-length, chromosomally encoded EspF and EspZ were used. After 2 h of infection, the C-terminal epitope tags from EspF and EspZ were identified within enriched mitochondria and EspF was detected at its MTS-cleaved molecular weight, suggesting mitochondrial localization and import (Fig. 1A). Similarly, ΔΨ_m_ was significantly decreased during wild-type EPEC infection compared with mock-infected cells, revealing the induction of early intrinsic apoptosis comparable with our earlier work capturing events before caspase-3 activation (*P < *0.0367) (Fig. 1B). Notably, the ΔΨ_m_ decrease during infection was not significantly different from that measured during chemically induced early intrinsic apoptosis with Bax agonist molecule 7 in our recent MS-TAILS study (*P* = 0.90) (16). Therefore, we used these conditions to compare the N-terminomes of infected cells and mitochondria during (i) mock infection, (ii) EPEC infection with the wild-type strain, and (iii) EPEC infection with a mutant strain lacking the EscN motor that drives type III secretion (Fig. 2).

### Infection altered the human cellular terminome.

For MS-TAILS, human epithelial HeLa cells were isotopically labeled with “light” (+0 Da) or “heavy” (+10 Da) arginine and infected (*n* = 3 biological replicates) (Fig. 2). Infected cells were pooled and 500 μg as retained for whole-cell TAILS analyses. Mitochondria were enriched, and the proteomes of whole-cell and mitochondrial fractions were isolated in parallel, taking needed precautions to prevent proteolysis during sample preparation. All primary amines were chemically blocked, including mature N termini and neo-N termini generated at proteolytic sites in each infection condition. Proteins were trypsinized, and the new, unblocked N termini were captured with a polyaldehyde-HPG-ALD polymer. The remaining N-terminally blocked peptides were identified and quantified by liquid chromatography tandem mass spectrometry (LC-MS/MS) using MaxQuant, the MitoCarta2.0 database of mitochondrial proteins, and our freely available MS-TAILS bioinformatics software suite (16, 19–21).

**FIG 3:**
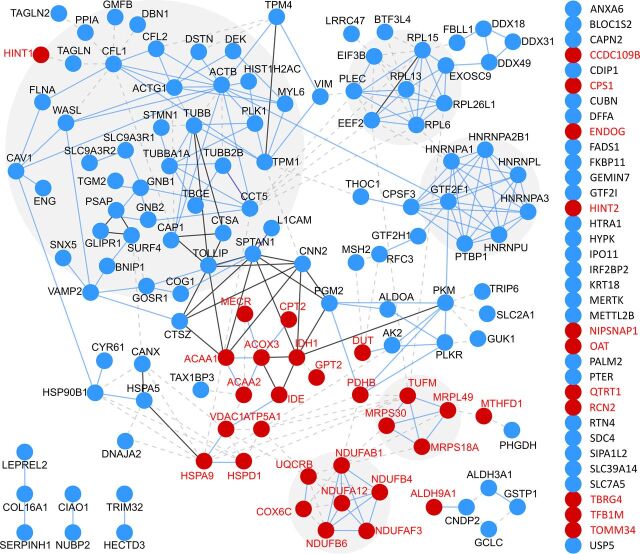
EPEC infection and T3S effectors altered N termini in 191 proteins across cellular and mitochondrial proteins. All proteins with significantly altered N-terminal peptides, in either cell fraction, were analyzed with STRING. Known mitochondrial proteins are displayed with red font. Blue circle, cellular protein; red circle, mitochondrial protein in MitoCarta2.0.

**FIG 4:**
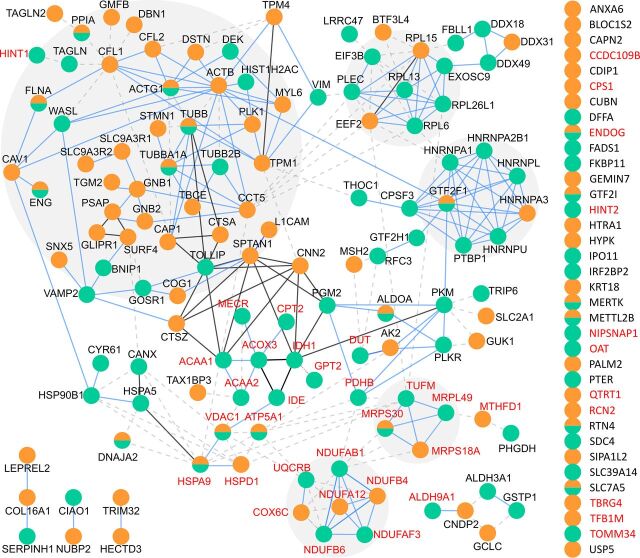
T3S effectors specifically altered a unique subset of N termini in cellular and mitochondrial proteins. All proteins with significantly altered N-terminal peptides were analyzed with STRING. Orange, infection dependent; green, T3S dependent. Known mitochondrial proteins are displayed with red font.

MS-TAILS identified 1,695 N-terminal peptides from 1,060 unique proteins at a false discovery rate (FDR) of 0.01 (Table 1; see Data Set S1 in the supplemental material). Of these peptides, the abundance of 230 N-terminal peptides was significantly different from EPEC wild-type infection (i.e., seen at least 1.5-fold more/less and with a *P* value of identification of <0.05) (see Fig. S1 in the supplemental material). Of all N-terminal peptide ratios altered during infection, those that significantly differed between wild-type versus mock infection were considered generally “infection dependent,” or those between wild-type versus *ΔescN* infection were considered specifically “T3S dependent.” These changes encompassed a network of 191 proteins across the human cellular and mitochondrial proteomes (Fig. 3), such as decreased initiator methionine (Met^1^) removal in the cell death-inducing p53 target protein during wild-type infection compared with uninfected cells. Of these proteins, 97 (50.8%) were identified with T3S-dependent changes (Fig. 4) and 40 (20.9%) were known mitochondrial proteins in the MitoCarta2.0 database (20, 21).

**TABLE 1:** MS-TAILS identified 1,060 unique proteins from 1,695 N-terminal peptides in whole cells and mitochondrial fractions^*a*^

MS-TAILS identification	Total (*n*)	Whole cell fraction (*n*)	Mitochondrial fraction (*n*)	Identified from mitochondrial vs. whole cell fraction (%)	Identified from whole cell fraction (% of total)
All proteins identified	1,060	738	474	64.2	69.6
All N-terminal peptides identified	1,695	1,010	737	73.0	59.6
Mitochondrial proteins identified	215	86	169	196.5	40.0
Mitochondrial N-terminal peptides identified	390	107	283	264.5	27.4

a Unique proteins and N-terminal peptides were identified from MS-TAILS of mitochondria and whole cells during EPEC infection experiments (*n* = 3; FDR = 0.01). Mitochondrial proteins were identified using the MitoCarta2.0 database (20, 21).

10.1128/mSystems.00283-20.1 FIG S1 EPEC infection resulted in quantitative changes in high-confidence N-terminal peptides from mitochondria and whole cells. The abundance of each identified N-terminal peptide from each cellular fraction is depicted in the EPEC wild-type (WT) infection condition relative to the mock-infected or EPEC Δ*escN* strain-infected conditions against the posterior error probability, or *P* value of identification. Green dots represent N-terminal peptides that were seen significantly more in the stated conditions (i.e., >1.5 fold more and with a *P* value of <0.05). Red dots represent those seen significantly less. Download FIG S1, TIF file, 2.4 MB. Copyright © 2020 Marshall et al. 2020 Marshall et al. This content is distributed under the terms of the Creative Commons Attribution 4.0 International license.

10.1128/mSystems.00283-20.3 DATA SET S1 N-terminal peptides identified from human cells infected with EPEC wild type or an EPEC T3S-deficient mutant or mock infected. Complete MS-TAILS dataset of human N-terminal peptides identified in this study as outlined in Fig. 2. N-terminal peptides were identified and quantified using MaxQuant (false discovery rate of 0.01) and annotated using the Terminal Annotation of Peptides (TAP) program to mine UniProt and TopFIND as previously described (16, 19). Mitochondrial proteins were identified with the MitoCarta2.0 database (20, 21). WT, wild-type EPEC; UI, uninfected; Δ*escN*, T3S-deficient strain of EPEC. Download Data Set S1, XLS file, 2.4 MB. Copyright © 2020 Marshall et al. 2020 Marshall et al. This content is distributed under the terms of the Creative Commons Attribution 4.0 International license.

### Infection altered termini within mitochondrial proteins.

**FIG 5:**
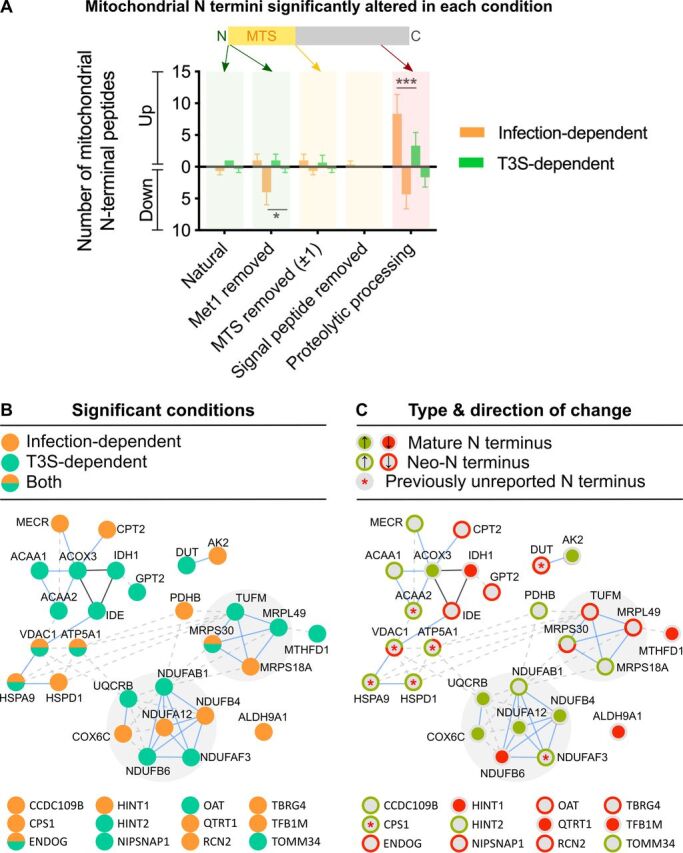
EPEC T3S effectors altered the human mitochondrial N-terminome. (A) Mitochondrial N-terminal peptides that were significantly increased or decreased during infection were annotated according to the N-terminal type indicated, including mature (i.e., at Met^1^, Met^1^ removal; green), transit peptide removal (yellow), and neo-N termini arising from proteolytic processing (red). Differences between the number of altered termini of each type were assessed with a two-way analysis of variance (ANOVA) with a Šídák multiple-comparison test (*, *P* < 0.05; ***, *P < *0.001). (B) The 40 mitochondrial proteins with infection- and/or T3-dependent changes (orange and green circles, respectively) were analyzed for protein-protein interactions using STRING. Blue line, binding partners; black line, reaction. (C) Each mitochondrial protein with a significant change in a mature N terminus is shown with a filled circle (green, significant increase; red, significant decrease). Those with a change in a neo-N terminus are shown with outer rings. Red asterisk (*), proteins containing significant neo-N termini at sites not previously reported.

MS-TAILS identified 390 N-terminal peptides from 215 mitochondrial proteins. The mitochondrial fraction identified 2.0-fold as many mitochondrial proteins and 2.6-fold as many mitochondrial N-terminal peptides as the whole cell, which identified only 27.4% of all mitochondrial N-terminal peptides overall (Table 1). Within the mitochondrial terminome, infection or T3S effectors altered the abundance of 40 mitochondrial proteins at 45 total sites, corresponding to either mature protein termini (Met^1^ or Met^1^ removal), an annotated MTS removal, signal peptide removal, or other proteolytic processing (Fig. 5A and B; see Table S1 in the supplemental material). Of these sites, 37.8% were T3S dependent (*n* = 17) (Fig. 5B) and 60.0% were neo-N termini from proteolytic processing (*n* = 27) (Fig. 5A and C), including 9 proteolytic sites not previously reported in the DegraBase or our prior MS-TAILS study of apoptosis, none of which occurred at classical DEVD caspase cleavage motifs (Table 2) (16, 22). Notably, there were no significant abundance changes observed in protein mature N termini, including MTS removal (Fig. 5A).

**TABLE 2:** Novel proteolytic sites identified in mitochondrial proteins during EPEC infection^*a*^

Gene	Protein name	N-terminal site^*b*^	Fold change, WT:mock		Fold change, WT:Δ*escN*		T3S dependent?
			C	M	C	M	
***ACAA2***	**3-Ketoacyl-CoA thiolase, mitochondrial**	KHKISR↓E^177*^		↑1.5			
***HSPD1***	**60-kDa heat shock protein, mitochondrial**	ALNATR↓A^430*^	ns	ns	ns	↑1.6	Yes
*ATP5A1*	ATP synthase subunit alpha, mitochondrial	SILEER↓I^59^^*^		ns		↑1.7	
*CPS1*	Carbamoyl-phosphate synthase [ammonia], mitochondrial	YPVMIR↓S^588^^*^		↑16.7			
*CPS1*	Carbamoyl-phosphate synthase [ammonia], mitochondrial	FLVKGN↓D^1250^^*^		↑2.3			
*DUT*	Deoxyuridine 5'-triphosphate nucleotidohydrolase, mitochondrial	MPC↓S^4^^*^	↓1.7	↓2.9	ns	ns	
*NDUFAF3*	NADH dehydrogenase [ubiquinone] 1 alpha subcomplex assembly factor 3	WAPRRG↓H^32^^*^	↑1.9	ns	ns	ns	
*HSPA9*	Stress-70 protein, mitochondrial	NAEGAR↓T^86^^*^		ns		↑2.2	Yes
***VDAC1***	**Voltage-dependent anion-selective channel protein 1**	TDNTLG↓T^83*^	↑10.1	↑3.4	↓1.7	↓1.7	Yes

a Mitochondrial N-terminal peptides that were altered in abundance during EPEC infection are listed according to the N-terminal type, site, and relative fold change between EPEC wild-type (WT) infection versus either mock-infected cells or T3S-deficient infection (Δ*escN*). These sites have not been previously reported in the DegraBase or MS-TAILS experiments of apoptosis (16, 22). C, whole-cell fraction; M, mitochondrial fraction; bold text, protein known to be associated with apoptosis from UniProt; ↓, N-terminal location of observed N terminus in the full-length protein; ns, not significantly altered in abundance; *, previously unreported site in the DegraBase or MS-TAILS of apoptosis (16, 22).

b N-terminal site denotes the six amino acids to the prime and nonprime sides of the predicted site of proteolysis (i.e., P6 to P6’).

10.1128/mSystems.00283-20.2 TABLE S1 EPEC infection and its T3S effectors altered the abundance of 45 N termini from 40 human mitochondrial proteins. Mitochondrial N-terminal peptides that were altered in abundance during EPEC infection are listed according to the N terminus type, site, and relative fold change between EPEC wild-type (WT) infection versus mock-infected cells and between WT versus T3S-deficient infection (Δ*escN*). Download Table S1, DOCX file, 0.04 MB. Copyright © 2020 Marshall et al. 2020 Marshall et al. This content is distributed under the terms of the Creative Commons Attribution 4.0 International license.

### Infection resulted in canonical apoptotic proteolytic changes in mitochondria.

**FIG 6:**
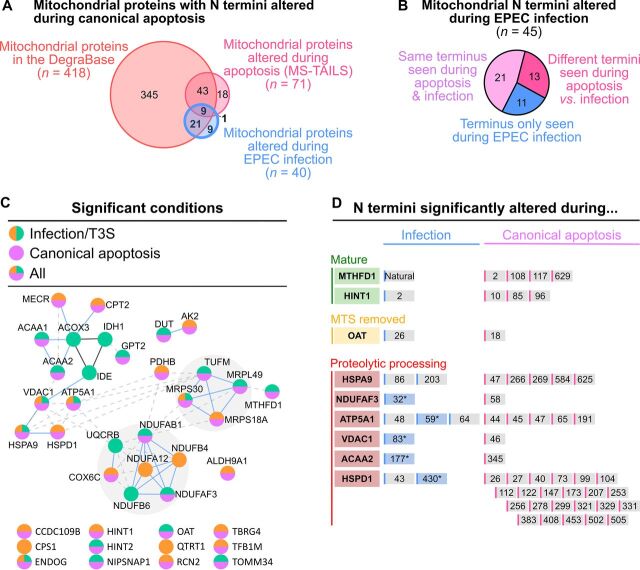
EPEC infection altered mitochondrial proteins involved with apoptosis. (A) Mitochondrial N-terminal peptides identified and quantified by MS-TAILS that showed significant alterations in abundance ratios during EPEC infection and/or in the presence of T3S effectors. Mitochondrial protein termini were compared with those altered during either chemical-induced intrinsic apoptosis with MS-TAILS or chemical-induced intrinsic or extrinsic apoptosis in the DegraBase (16, 22). A common subset of 31 proteins was identified. (B) N termini abundances that were affected in an infection- or T3S-dependent manner were compared with those altered in prior apoptosis studies (pink). (C) The sites of infection-/T3S-dependent N termini in mitochondrial proteins (green and orange, respectively) were compared with the DegraBase and MS-TAILS. A common subset of 21 N termini was identified. (D) Thirteen infection-/T3S-dependent mitochondrial termini were altered in proteins also seen in prior studies of apoptosis. However, the sites within each protein were different, including 2 mature protein N termini, 1 MTS removal N terminus, and 10 proteolytic processing termini in 6 proteins. The P1’ amino acid position of significantly altered N termini are indicated. Previously unreported termini are displayed with a blue background.

To identify known mitochondrial changes due to apoptosis, we compared the 45 mitochondrial termini altered during infection with those identified in (i) the DegraBase apoptosis terminomics database, and (ii) our MS-TAILS study of chemical-induced, early intrinsic apoptosis of HeLa cells (Fig. 6) (16, 22). Of the 40 mitochondrial proteins altered during infection, 31 (77.5%) were also altered during chemical apoptosis, suggesting similar apoptotic events during both chemical induction and EPEC infection (Fig. 6A and B). Of the 45 mitochondrial termini altered during infection, 21 (46.7%) were also altered in chemical apoptosis (Fig. 6C), including T3S-dependent neo-N termini in three proteins involved in apoptosis, namely, endonuclease G (MTS removal), 39S ribosomal protein S30 (P1’ = Ala^26^; i.e., proteolysis to the N terminus of Ala^26^), and histidine triad nucleotide-binding protein 2 (P1’ = Ala^31^) (Table 3).

**TABLE 3:** Twenty-one mitochondrial protein termini were significantly altered in abundance during both EPEC infection and apoptosis^*a*^

Gene by terminus type	Protein name	N terminus site (P6–P1’)	T3S dependent?
Natural protein terminus			
*AK2*	Adenylate kinase 2, mitochondrial	M^1^	Yes
*COX6C*	Cytochrome *c* oxidase subunit 6C	M^1^	Yes
Initiator methionine removed			
*ALDH9A1*	4-Trimethylaminobutyraldehyde dehydrogenase	M↓S^2^	
*TFB1M*	Dimethyladenosine transferase 1, mitochondrial	M↓A^2^	
MTS removed			
*NDUFAB1*	Acyl carrier protein, mitochondrial	QLCRQY↓S^69^	
*CPT2*	Carnitine O-palmitoyltransferase 2, mitochondrial	APSRPL↓S^26^	
***ENDOG***	**Endonuclease G, mitochondrial**	**LPVAAA↓A^49^**	**Yes**
Other signal peptide removed			
*RCN2*	Reticulocalbin-2	CAAAAG↓A^22^	
Proteolytic processing			
*MRPL49*	39S ribosomal protein L49, mitochondrial	CGLRLL↓S^27^	
*MRPS18A*	39S ribosomal protein S18a, mitochondrial	RLPARG↓F^35^	Yes
***MRPS30***	**39S ribosomal protein S30, mitochondrial**	**TAANAA↓A^26^**	**Yes**
*ACAA1*	3-Ketoacyl-CoA thiolase, peroxisomal	PQAAPC↓L^27^	
*GPT2*	Alanine aminotransferase 2	SWGRSQ↓S^25^	
*CCDC109B*	Calcium uniporter regulatory subunit MCUb, mitochondrial	YQSHHY↓S^52^	
*TUFM*	Elongation factor Tu, mitochondrial	LLDAVD↓T^245^	
*MECR*	Enoyl-[acyl-carrier-protein] reductase, mitochondrial	GCHGPA↓A^31^	
***HINT2***	**Histidine triad nucleotide-binding protein 2, mitochondrial**	**GGQVRG↓A^31^**	**Yes**
*TOMM34*	Mitochondrial import receptor subunit TOM34	MAP↓K^4^	Yes
*NIPSNAP1*	Protein NipSnap homolog 1	AAAARF↓Y^35^	Yes
*TBRG4*	Protein TBRG4	VAHKTL↓T^40^	
*PDHB*	Pyruvate dehydrogenase E1 component subunit beta, mitochondrial	LQVTVR↓D^37^	

a EPEC infection- and T3S-dependent mitochondrial N termini were compared with those from our previous MS-TAILS study of chemical-induced early intrinsic apoptosis and with termini identified in the DegraBase database of apoptotic protein termini (16, 22). Bold text, protein known to be associated with apoptosis; ↓, N-terminal location of observed N terminus in the full-length protein.

### Infection uniquely altered the abundance of N termini from proteins annotated as being involved in apoptosis.

To identify infection-specific or candidate T3S-mediated events, we examined the 24 mitochondrial termini that had not been identified previously during canonical apoptosis. Nine proteins were known to be cleaved during apoptosis but not at the terminus observed during infection, which therefore represents alternative cleavage sites in these classically targeted proteins. For five proteins, the terminus was at a previously unreported site, and three of these proteins have a known role in apoptosis, namely, voltage-dependent anion-selective channel protein 1 (VDAC1), 60-kDa heat shock protein 1 (HSPD1), and ATP synthase subunit alpha (ATP5A1) (Fig. 6D; Table 4). These cleavage events may be due to alternative apoptotic pathways triggered by EPEC effectors.

**TABLE 4:** Mitochondrial protein termini altered during infection but not in prior apoptosis studies^*a*^

Terminus type	Gene by terminus type	Protein name	N terminus site (P6–P1’)	T3S dependent?
Termini from proteins cleaved at different sites in prior apoptosis studies	Natural protein terminus			
	*MTHFD1*	C-1-tetrahydrofolate synthase, cytoplasmic	M^1^	Yes
	Initiator methionine removed			
	***HINT1***	**Histidine triad nucleotide-binding protein 1**	M↓A^2^	
	MTS removed			
	*OAT*	Ornithine aminotransferase, mitochondrial	SSVASA↓T^26^	Yes
	Proteolytic processing			
	***ACAA2***	**3-Ketoacyl-CoA thiolase, mitochondrial**	KHKISR↓E^177^*	
	***HSPD1***	**60-kDa heat shock protein, mitochondrial**	**RALMLQ↓G^43^**	**Yes**
	***HSPD1***	**60-kDa heat shock protein, mitochondrial**	ALNATR↓A^430^*	**Yes**
	*ATP5A1*	ATP synthase subunit alpha, mitochondrial	HLQKTG↓T^48^	Yes
	*ATP5A1*	ATP synthase subunit alpha, mitochondrial	SILEER↓I^59^*	
	*ATP5A1*	ATP synthase subunit alpha, mitochondrial	RILGAD↓T^64^	
	*NDUFAF3*	NADH dehydrogenase [ubiquinone] 1 alpha subcomplex assembly factor 3	WAPRRG↓H^32^*	
	***HSPA9***	**Stress-70 protein, mitochondrial**	NAEGAR↓T^86^*	**Yes**
	***HSPA9***	**Stress-70 protein, mitochondrial**	**FNDSQR↓Q^203^**	
	***VDAC1***	**Voltage-dependent anion-selective channel protein 1**	TDNTLG↓T^83^*	**Yes**
Termini that were observed during infection but not in prior apoptosis studies	Natural protein terminus			
	*NDUFA12*	NADH dehydrogenase [ubiquinone] 1 alpha subcomplex subunit 12	M^1^	Yes
	*NDUFB6*	NADH dehydrogenase [ubiquinone] 1 beta subcomplex subunit 6	M^1^	
	Met1 removed			
	*UQCRB*	Cytochrome b-c1 complex subunit 7	M↓A^2^	
	*IDH1*	Isocitrate dehydrogenase [NADP] cytoplasmic	M↓S^2^	
	*NDUFB4*	NADH dehydrogenase [ubiquinone] 1 beta subcomplex subunit 4	M↓S^2^	Yes
	*ACOX3*	Peroxisomal acyl-coenzyme A oxidase 3	M↓A^2^	
	*QTRT1*	Queuine tRNA-ribosyltransferase catalytic subunit 1	M↓A^2^	
	Proteolytic processing			
	*CPS1*	Carbamoyl-phosphate synthase [ammonia], mitochondrial	YPVMIR↓S^588^*	
	*CPS1*	Carbamoyl-phosphate synthase [ammonia], mitochondrial	FLVKGN↓D^1250^*	
	*DUT*	Deoxyuridine 5'-triphosphate nucleotidohydrolase, mitochondrial	MPC↓S^4^*	
	*IDE*	Insulin-degrading enzyme	KKTYSK↓M^42^	

a Mitochondrial N termini that were significantly altered during infection were compared with known apoptotic termini from the DegraBase or MS-TAILS, as well as the Merops peptidase database, to compare infection- and T3S-dependent termini with known proteolytic events in human proteins (16, 22, 23). Bold text, protein known to be associated with apoptosis.; ↓, N-terminal location of observed N terminus in the full-length protein; *, previously unreported site in the DegraBase, MS-TAILS of apoptosis, or Merops (16, 22, 23).

### Novel mitochondrial neo-N termini were observed during infection.

The remaining 11 mitochondrial N termini that were not identified during canonical apoptosis were also not associated with apoptosis in earlier studies, including 7 mature N termini and 4 neo-N termini. Three of four neo-N termini were previously unreported in prior terminomics studies or in Merops, namely, carbamoyl-phosphate synthase (ammonia) (P1’ = Ser^588^ and Asp^1250^) and deoxyuridine 5′-triphosphate nucleotidohydrolase (P1’ = Ser^4^) (Table 4) (23), implying the presence of novel proteolytic sites in these proteins in the context of infection.

## DISCUSSION

In this study, we demonstrate that both T3S-dependent and -independent processes occur in mitochondria during EPEC infection. Of all 230 N termini altered in abundance during infection, 45 were from mitochondrial proteins and approximately half were T3S dependent. We show that nearly half of EPEC-induced mitochondrial events were directly attributable to canonical apoptosis, suggesting that most mitochondrial changes during infection may be from nonapoptotic virulence mechanisms. Furthermore, we identify nine mitochondrial neo-N termini not previously reported in healthy or apoptotic cells. As the first direct N-terminomics study of bacterial infection, this work provides the first look at global protease dysregulation during active infection and a novel perspective of T3S-mediated disease during infection.

During EPEC infection, multiple virulence factors traffic to mitochondria and promote or delay apoptosis. However, the underlying mechanisms are unknown. Systems-level approaches have proven insightful for deciphering the complex interaction between a pathogen and its host during infection (15, 24). Due to the key roles of proteolysis in inflammation and the immune response (both frequent targets of virulence factors) terminomics is a powerful approach for deciphering pathogenic mechanisms involving proteolysis. Our previous N-terminomics analyses using TAILS identified viral and human protease substrates from cell lysates but not from active infection (25–28). Thus, we applied mitochondrial terminomics to identify T3S-dependent changes and, thereby, assess how effectors manipulate apoptosis while capturing the host response.

Due to the crucial role of proteolysis in apoptosis, it was necessary to distinguish canonical apoptotic events in order to identify those that arose due to bacterial virulence or host defense. Most of the significant changes in the mitochondrial terminome were in neo-N termini, demonstrating altered proteolytic processing of many mitochondrial proteins during infection that occurred with few changes in mature protein N termini that would otherwise suggest a concurrent change in protein abundance. We show that nearly half of altered mitochondrial termini that were altered in abundance during infection were identical to those seen in canonical apoptosis, including that in our earlier MS-TAILS study of mitochondria during early intrinsic apoptosis in the same cell line as well as in whole-cell terminome studies of intrinsic and extrinsic apoptosis in multiple cell lines in the DegraBase (16, 22). Mitochondrial changes that occurred during both infection and apoptosis included established apoptotic events, such as MTS removal of endonuclease G and proteolysis in histidine triad nucleotide-binding protein 2 (Hint2; P1’ = Ala^31^), an apoptotic sensitizer correlated with increased rates of cell death following apoptosis initiation (29). Mitochondrial changes that were not previously reported from apoptosis may inform how mitochondrial-targeting T3S effectors modulate cell death, such as an alternative strategy to subvert host-regulated apoptosis.

In particular, eight mitochondrial proteins were identified with novel neo-N termini or proteolysis, namely, VDAC1, HSPD1, HSPA9, ACAA2, ATP5A1, NDUFAF3, CPS1, and DUT (Table 2). None of these novel mitochondrial cleavage sites occurred at DEVD cleavage motifs, which are classically associated with caspases, and may instead indicate the involvement of an EPEC protease within mitochondria, the dysregulation of a human mitochondrial protease, or the induction of a human protease that modulates apoptosis in the context of infection. For instance, a previously unreported neo-N terminus in VDAC1 (P1’ = Thr^83^) was altered in an infection- and T3S-dependent manner. Proteolysis at this site could directly impact the formation of the VDAC1-containing pore in the inner mitochondrial membrane, through which toxic, proapoptotic proteins are released, accelerating cell-wide consequences of apoptosis. Second, a previously unreported neo-N terminus in HSPD1 (P1’ = Ala^430^) could abrogate the function of this mitochondrial chaperone, which was recently shown to modulate cellular immune pathways and resistance to Pseudomonas aeruginosa infection (30). Finally, proteolysis in ACAA2 (P1’ = Glu^177^) would physically divide its active site and potentially abrogate its binding to a proapoptotic protein in the Bcl-2 family that is also altered in Shigella flexneri infection, namely, BCL2/adenovirus E1B 19-kDa protein-interacting protein 3 (BNIP3) (31). ACAA2 binding abrogates BNIP3-mediated apoptosis (32); therefore, increased ACAA2 proteolysis may overall promote BNIP3-mediated apoptosis during infection.

While our study focused on mitochondria, MS-TAILS also identified changes in the whole-cell N-terminome as well as changes in proteins that cofractionated with mitochondria. These N-terminomes may shed light upon important differences in mitochondrial protein import and protein-protein interactions, particularly with recent evidence from Scott et al. about the importance of the mitochondrial membrane interactome in apoptosis (33). By combining both mitochondrial and whole-cell changes, MS-TAILS depicts a broader view of affected pathways and may help to contextualize interesting findings. For example, decreased initiator Met removal in the cell death-inducing p53 target protein during infected versus mock-infected cells suggests a decreased abundance of this protein that regulates apoptosis mediated through tumor necrosis factor α (34). Similar approaches may be particularly useful in examining infection with pathogens that alter cytosolic proteolysis, including both EPEC and the intracellular pathogen Shigella flexneri, which inhibit cytosolic caspases during infection using the T3S effector NleF and cytosolic lipopolysaccharide, respectively (35, 36).

Ultimately, N-terminomic approaches are an important complement to build complete host-pathogen protein interaction networks and fully understand host-pathogen interactions (37–40), despite the many challenges in studying this complex and dynamic association (reviewed by Fels et al. [41]). This study demonstrates the potential of organelle-specific N-terminomics in studying infection for understanding how pathogens target and disrupt mitochondrial signaling. Hence, using TAILS to identify new molecular events during infection will improve our understanding of how and why multiple EPEC T3S effectors localize to mitochondria and subvert apoptosis. Subsequent terminomic studies with EPEC strains lacking single T3S effectors (e.g., Δ*espF* and Δ*espZ*) should help identify the specific roles of each effector during T3S-mediated subversion of apoptosis. Ultimately, temporal and *in vivo* studies may be essential to distill the role of each effector and their complex interplay in host immune suppression and apoptosis of intestinal epithelial cells along microvilli before shedding and transmission to the next host.

Because many important human pathogens possess virulence factors that target mitochondria and apoptosis (6), similar approaches can help us understand how many pathogens manipulate apoptosis during infection. For instance, several pathogens identified as priority threats for antimicrobial resistance by the U.S. Centers for Disease Control of Prevention also target mitochondrial signaling pathways (e.g., Acinetobacter baumannii, Clostridioides difficile, Mycobacterium tuberculosis, Neisseria gonorrhoeae, Staphylococcus aureus, and many *Enterobacteriaceae* members), suggesting the broad and applicable value of MS-TAILS for translational benefits to human health (42).

## MATERIALS AND METHODS

### Cell culture.

HeLa cells (CCL-2, American Type Culture Collection) were cultured in Dulbecco’s modified Eagle medium (DMEM; HyClone) with high glucose and sodium pyruvate supplemented with heat-inactivated fetal bovine serum (10% vol/vol; Gibco), GlutaMax (1% vol/vol; HyClone), and nonessential amino acids (1% vol/vol; HyClone). Cells were used between passages 5 and 20.

### Infection.

The following four EPEC O127:H6 E2348/69 strains were used: wild type, Δ*escN*, Δ*espZ*/*espZ:HA*, and Δ*espF*/*espF:HSV* (10, 43). HeLa human epithelial cells (2.0 × 10^6^) were seeded into 15-cm tissue culture plates and allowed to grow to 75% confluence. EPEC colonies were incubated in lysogeny broth overnight, and T3S was preinduced by subculturing 1:20 in prewarmed DMEM without phenol red (G.E. Healthcare) at 37°C in 5% CO_2_ for 3.5 h without shaking. Simultaneously, HeLa cells were synchronized in prewarmed, serum-free DMEM for 3 h. A replicate 15-cm plate of HeLa cells was trypsinized and counted to determine the number of cells to be infected, and the volume of preinduced culture required for a multiplicity of infection of 20:1 was calculated at optical density at 600 nm (OD_600_). For infection, HeLa cell medium was replaced with 15 ml of prewarmed, serum-free DMEM containing preinduced bacteria and incubated for 2 h. Mock infections were performed only with DMEM lacking phenol red.

### Mitochondrial membrane potential (ΔΨ_m_) assay.

HeLa cells (5 × 10^4^) were seeded into each well of a 96-well plate (Costar) and incubated overnight at 37°C with 5% CO_2_. Prepared JC-1 dye was added to each well 15 min before the end of infection. ΔΨ_m_ was measured according to the manufacturer’s instructions (Cayman Chemical) on a Tecan M200 plate reader. J-aggregate:J-monomer ratios were normalized to the relevant control (i.e., mock-infected cells for infected cells and vehicle control-treated cells for Bax agonist molecule 7; Calbiochem). Three technical replicates for each of three independent biological replicates were performed for each condition.

### Mitochondrial enrichment.

Cells were lysed manually in a prechilled glass Teflon Potter-Elvehjem homogenizer on ice, and mitochondrial enrichments were performed by sequential centrifugation in a sucrose-containing buffer with protease inhibitors (5 mM EDTA and EDTA-free HALT protease inhibitor cocktail; Thermo Fisher), as described previously by Frezza et al. and adapted by Marshall et al. (16, 44).

### Western blotting.

Wells of 15% SDS-polyacrylamide gels were loaded with 20 μl of protein sample and analyzed by SDS-PAGE and Western blotting as previously described (16). Membranes were incubated overnight with the primary antibody in blocking buffer at 4°C with a primary antibody, namely, anti-cytochrome *c* oxidase IV (COX-IV; 1:1,000; 3E11; Cell Signaling Technologies), anti-calnexin (1:1,000; Enzo Life Sciences), anti-β-tubulin (1:5,000; number T4026; Sigma-Aldrich), anti-herpes simplex virus epitope tag (1:1,000; Abcam), anti-hemagglutinin (1:1,000; Roche), anti-ACAA2 (1:1,000; Abcam), anti-COX6C (1:1,000; Abcam), anti-DNAJA3 (1:500; Abcam), or anti-endonuclease G (1:500; Abcam).

### MS-TAILS N-terminomics.

Two populations of HeLa cells were cultured separately in high-glucose DMEM without arginine and lysine (Caisson Labs) supplemented with l-lysine (1.0 M; Sigma-Aldrich), dialyzed fetal bovine serum (10% vol/vol; Gibco), GlutaMax (1% vol/vol), and nonessential amino acids (1% vol/vol). For media for each stable isotope labeling by amino acids in cell culture (SILAC) cell population, arginine was replaced with either normal or heavy isotope-coded arginine (^13^C(6), ^15^N(4) [arginine]; Cambridge Isotope Laboratories). Two parallel MS-TAILS experiments were prepared, as in Fig. 2. Following infection or mock infection, cells were pooled 1:1 and lysed, and mitochondria were enriched. Three independent biological replicates were performed on separate days at subsequent cell passages. For each replicate, 500 μg of whole-cell lysate and 500 μg of enriched mitochondria were analyzed in parallel. MS-TAILS was performed as previously described (16). Briefly, the collected proteome was precipitated with chloroform-methanol and the precipitate was reconstituted in 1 M guanidine chloride in HEPES (pH 7.5). Proteins were denatured and reduced with 10 mM DTT at 60°C, and subsequently, cysteines were alkylated with 15 mM iodoacetamide in the dark. After pH adjustment to 6.5, free amine groups were blocked by reductive amination with 40 mM formaldehyde using cyanoborohydride as a catalyst for 18 h at 37°C. After sample cleanup by chloroform-methanol precipitation, proteins were digested with trypsin, and N-terminal peptides were negatively enriched using 2.5 mg of a soluble aldehyde-functionalized highly-branched polyglycerol, aldehyde-derivatized (HPG-ALD) polymer (http://flintbox.com/public/project/1948/).

### Mass spectrometry.

MS-TAILS samples were analyzed on a linear-trapping quadrupole-Orbitrap Velos tandem mass spectrometer (ThermoFisher Scientific) following a 90-minute high-performance liquid chromatography (HPLC) gradient (Agilent 1290; ThermoFisher Scientific), as previously described (45).

### Bioinformatics.

N-terminomics data analysis was performed at a false discovery rate of 0.01 using MaxQuant version 1.5.2.8 (19) and the UniProt/Swiss-Prot human protein database (version 2013_10; 84,843 entries) as previously described (16). Mitochondrial proteins were identified with the MitoCarta2.0 database (20, 21). Protein interaction networks were assembled using STRING (version 11.0) with a minimum interaction score of 0.700 at high confidence (46).

### Data availability.

The mass spectrometry proteomics data have been deposited in the ProteomeXchange Consortium via the PRIDE partner repository with the data set identifier PXD016994 (47).
