# Supplementary material for: Master Sculptor at Work: Enteropathogenic Escherichia coli Infection Uniquely Modifies Mitochondrial Proteolysis during Its Control of Human Cell Death
Source: mSystems. 2020 Jun 2;5(3):e00283-20. doi: 10.1128/mSystems.00283-20 (PMC8534729; doi:10.1128/mSystems.00283-20)
Supplement: TABLE S1 [file msystems.00283-20-st001.docx]

**TABLE S1.**

|  | **Gene** | **Protein name** | **N terminus site^b^** | **Fold change WT:mock** | | **Fold change WT:ΔescN** | | |  |
| --- | --- | --- | --- | --- | --- | --- | --- | --- | --- |
|  |  |  |  | **C** | **M** | | **C** | **M** | |
| **Natural protein terminus** | | | | | | | | | |
|  | *AK2* | Adenylate kinase 2, mitochondrial | M^1^ | ns | ns | | ns | **↑1.5** | |
|  | *MTHFD1* | C-1-tetrahydrofolate synthase, cytoplasmic | M^1^ | ns | ns | | **↓2.2** | ns | |
|  | *COX6C* | Cytochrome *c* oxidase subunit 6C | M^1^ | ns | ns | | ns | **↑1.7** | |
|  | *NDUFA12* | NADH dehydrogenase [ubiquinone] 1 alpha subcomplex subunit 12 | M^1^ | ns | ns | | **↑2.0** | ns | |
|  | *NDUFB6* | NADH dehydrogenase [ubiquinone] 1 beta subcomplex subunit 6 | M^1^ | ns | **↓2.0** | | ns | ns | |
| **Initiator methionine removed** | | | | | | | | | |
|  | *ALDH9A1* | 4-trimethylaminobutyraldehyde dehydrogenase | M↓S^2^ | ns | **↓2.7** | | ns | ns | |
|  | *UQCRB* | Cytochrome b-c1 complex subunit 7 | M↓A^2^ | **↑3.8** | ns | | ns | ns | |
|  | *TFB1M* | Dimethyladenosine transferase 1, mitochondrial | M↓A^2^ | **↓5.6** | **↓1.7** | | ns | ns | |
|  | *HINT1* | Histidine triad nucleotide-binding protein 1 | M↓A^2^ | ns | **↓1.5** | | ns | ns | |
|  | *IDH1* | Isocitrate dehydrogenase [NADP] cytoplasmic | M↓S^2^ | ns | **↓1.6** | | ns | ns | |
|  | *NDUFB4* | NADH dehydrogenase [ubiquinone] 1 beta subcomplex subunit 4 | M↓S^2^ | ns | ns | | **↑1.5** | **↑1.6** | |
|  | *ACOX3* | Peroxisomal acyl-coenzyme A oxidase 3 | M↓A^2^ | ns | **↑1.8** | | ns | ns | |
|  | *QTRT1* | Queuine tRNA-ribosyltransferase catalytic subunit 1 | M↓A^2^ | ns | **↓3.0** | | ns | ns | |
| **MTS removed** | | | | | | | | | |
|  | *NDUFAB1* | Acyl carrier protein, mitochondrial | QLCRQY↓S^69^ | **↑2.1** | **↑1.7** | | ns | ns | |
|  | *CPT2* | Carnitine O-palmitoyltransferase 2, mitochondrial | APSRPL↓S^26^ | **↓1.7** | ns | | ns | ns | |
|  | *ENDOG* | Endonuclease G, mitochondrial | LPVAAA↓A^49^ | ns | **↓1.6** | | **↓4.2** | ns | |
|  | *OAT* | Ornithine aminotransferase, mitochondrial | SSVASA↓T^26^ | ns | ns | | ns | **↓1.6** | |
| **Signal peptide removed** | | | | | | | | | |
|  | *RCN2* | Reticulocalbin-2 | CAAAAG↓A^22^ | **↓1.7** | ns | | ns | ns | |
| **Proteolytic processing** | | | | | | | | | |
|  | *MRPL49* | 39S ribosomal protein L49, mitochondrial | CGLRLL↓S^27^ | **↓2.4** | **↓1.9** | | ns | ns | |
|  | *MRPS18A* | 39S ribosomal protein S18a, mitochondrial | RLPARG↓F^35^ | ns | ns | | ns | **↑1.9** | |
|  | *MRPS30* | 39S ribosomal protein S30, mitochondrial | TAANAA↓A^26^ | ns | **↓1.5** | | ns | **↑1.7** | |
|  | *ACAA2* | 3-ketoacyl-CoA thiolase, mitochondrial | KHKISR↓E^177^**^*^** | ns | **↑1.5** | | ns | ns | |
|  | *ACAA1* | 3-ketoacyl-CoA thiolase, peroxisomal | PQAAPC↓L^27^ | **↑3.6** | **↑1.6** | | ns | ns | |
|  | *HSPD1* | 60 kDa heat shock protein, mitochondrial | RALMLQ↓G^43^ | ns | ns | | **↑1.7** | ns | |
|  | *HSPD1* | 60 kDa heat shock protein, mitochondrial | ALNATR↓A^430^**^*^** | ns | ns | | ns | **↑1.6** | |
|  | *GPT2* | Alanine aminotransferase 2 | SWGRSQ↓S^25^ | ns | **↓1.5** | | ns | ns | |
|  | *ATP5A1* | ATP synthase subunit alpha, mitochondrial | HLQKTG↓T^48^ | **↓2.0** | ns | | ns | ns | |
|  | *ATP5A1* | ATP synthase subunit alpha, mitochondrial | SILEER↓I^59^**^*^** | ns | ns | | ns | **↑1.7** | |
|  | *ATP5A1* | ATP synthase subunit alpha, mitochondrial | RILGAD↓T^64^ | **↑1.5** | ns | | ns | ns | |
|  | *CCDC109B* | Calcium uniporter regulatory subunit MCUb, mitochondrial | YQSHHY↓S^52^ | **↑2.1** | ns | | ns | ns | |
|  | *CPS1* | Carbamoyl-phosphate synthase [ammonia], mitochondrial | YPVMIR↓S^588^**^*^** | ns | **↑16.7** | | ns | ns | |
|  | *CPS1* | Carbamoyl-phosphate synthase [ammonia], mitochondrial | FLVKGN↓D^1250^**^*^** | ns | **↑2.3** | | ns | ns | |
|  | *DUT* | Deoxyuridine 5'-triphosphate nucleotidohydrolase, mitochondrial | MPC↓S^4^**^*^** | **↓1.7** | **↓2.9** | | ns | ns | |
|  | *TUFM* | Elongation factor Tu, mitochondrial | LLDAVD↓T^245^ | **↓2.2** | ns | | ns | ns | |
|  | *MECR* | Enoyl-[acyl-carrier-protein] reductase, mitochondrial | GCHGPA↓A^31^ | **↑1.5** | ns | | ns | ns | |
|  | *HINT2* | Histidine triad nucleotide-binding protein 2, mitochondrial | GGQVRG↓A^31^ | ns | ns | | **↑1.7** | **↑1.6** | |
|  | *IDE* | Insulin-degrading enzyme | KKTYSK↓M^42^ | ns | **↓1.6** | | ns | ns | |
|  | *TOMM34* | Mitochondrial import receptor subunit TOM34 | MAP↓K^4^ | ns | ns | | **↑1.9** | ns | |
|  | *NDUFAF3* | NADH dehydrogenase [ubiquinone] 1 alpha subcomplex assembly factor 3 | WAPRRG↓H^32^**^*^** | **↑1.9** | ns | | ns | ns | |
|  | *NIPSNAP1* | Protein NipSnap homolog 1 | AAAARF↓Y^35^ | ns | ns | | **↓2.0** | **↓2.0** | |
|  | *TBRG4* | Protein TBRG4 | VAHKTL↓T^40^ | ns | **↓1.7** | | ns | ns | |
|  | *PDHB* | Pyruvate dehydrogenase E1 component subunit beta, mitochondrial | LQVTVR↓D^37^ | ns | **↑1.5** | | ns | ns | |
|  | *HSPA9* | Stress-70 protein, mitochondrial | NAEGAR↓T^86^**^*^** | ns | ns | | ns | **↑2.2** | |
|  | *HSPA9* | Stress-70 protein, mitochondrial | FNDSQR↓Q^203^ | ns | **↑1.7** | | ns | ns | |
|  | *VDAC1* | Voltage-dependent anion-selective channel protein 1 | TDNTLG↓T^83^**^*^** | **↑10.1** | **↑3.4** | | **↓1.7** | **↓1.7** | |
